# Evaluation of the functional effects of genetic variants‒missense and nonsense SNPs, indels and copy number variations‒in the gene encoding human deoxyribonuclease I potentially implicated in autoimmunity

**DOI:** 10.1038/s41598-019-49935-y

**Published:** 2019-09-20

**Authors:** Misuzu Ueki, Kaori Kimura-Kataoka, Junko Fujihara, Reiko Iida, Yasuyuki Kawai, Akari Kusaka, Takamitsu Sasaki, Haruo Takeshita, Toshihiro Yasuda

**Affiliations:** 1 Department of Medical Genetics and Biochemistry, Faculty of Medical Sciences, University of Fukui, Eiheiji, Fukui Japan; 2 Department of Legal Medicine, Shimane University School of Medicine, Enya, Izumo Japan; 3 Department of Life Sciences, Faculty of Medical Sciences, University of Fukui, Eiheiji, Fukui Japan; 4 Department of Cardiology, Kanazawa Medical University, Uchinada, Ishikawa Japan

**Keywords:** Genetics, Medical genetics

## Abstract

Genetic variants, such as single nucleotide polymorphisms (SNPs), in the deoxyribonuclease I (DNase I) gene which remarkably reduce or abolish the activity are assumed to be substantially responsible for the genetic backgrounds determining susceptibility to autoimmune dysfunction. Here, we evaluated many genetic variants, including missense and nonsense SNPs, and indel (inframe) variants in the gene, potentially implicated in autoimmune diseases as functional variants resulting in altered activity levels. Eighteen missense and 7 nonsense SNPs, and 9 indel (inframe) variants were found to result in loss of function and disappearance of DNase I activity. Furthermore, considering the positions in the DNase I protein corresponding to the various nonsense SNPs, all of the other nonsense SNPs and frameshift variants registered in the Ensembl database (https://asia.ensembl.org) appear likely to exert a pathogenetic effect through loss of the activity. Accordingly, a total of 60 genetic variants in the DNase 1 gene (*DNASE1*) inducing abolishment or marked reduction of the DNase I activity could be identified as genetic risk factors for autoimmunity, irrespective of how sparsely they were distributed in the population. It was noteworthy that SNP p.Gln244Arg, reportedly associated with autoimmunity and reducing the activity to about half of that of the wild type, and SNP p.Arg107Gly, abolishing the activity completely, were distributed worldwide and in African populations at the polymorphic level, respectively. On the other hand, with regard to copy number variations in *DNASE1* where loss of copy leads to a reduction of the *in vivo* enzyme activity, only 2 diploid copy numbers were distributed in Japanese and German populations, demonstrating no loss of copy. These exhaustive data for genetic variants in *DNASE1* resulting in loss or marked reduction of the DNase I activity are highly informative when considering genetic predisposition leading to autoimmune dysfunction.

## Introduction

Deoxyribonuclease (DNase)-mediated clearance of cell debris resulting from apoptosis and/or necrosis has been suggested to be primarily implicated in the prevention of autoimmune diseases such as systemic lupus erythematosus (SLE)^1,2^. Moreover, recent studies have demonstrated that extracellular DNase could serve as an endogenous regulator of neutrophil extracellular traps (NETs) and might improve the flow through DNase-dependent thrombolysis, thus preventing downstream injury^3,4^.

In the context of autoimmunity, serum DNase I, together with DNase I-like 3 (1L3), has been postulated to break down chromatin during apoptosis and/or necrosis^5–7^. DNase I-deficient mice have been reported to develop SLE-like syndromes^8^. Novel nonsense (p. Lys5*) and missense (p.Val111Met) SNPs in *DNASE1* have been identified in patients with autoimmune disease^9,10^. Previous studies have indicated that the frequency of the homozygote for the *G* allele in SNPp.Arg244Gln in *DNASE1* is much higher in patients with SLE who possess the corresponding autoantibodies than in patients who do not^11^. Furthermore, it has been demonstrated that the same allele in the corresponding SNP, leading to decrease in *in vivo* DNase I activity, is associated with a higher incidence of myocardial infarction (MI)^12^. These findings strongly suggest that genetic variants in *DNASE1* resulting in reduction or loss of *in vivo* DNase I activity might be substantially involved in the genetic backgrounds determining susceptibility to such diseases.

Many genetic variants in human *DNASEI* have been screened and registered on the Ensembl database (https://asia.ensembl.org; January 2018). Among them, we have been focusing on missense SNPs in *DNASEI* that are likely to affect the DNase I activity through the corresponding amino acid substitution, possibly serving as a pathogenetic background factor^12–16^. Since then, a large number of missense SNPs that would likely affect the catalytic activity have been newly identified. Furthermore, 19 nonsense SNPs, and 16 insertion/deletion (indel) (inframe) variants in *DNASE1* are evident in the Ensembl database. However, it remains to be clarified whether these 95 genetic variants could affect the DNase I activity.

It is estimated that chromosomal regions influenced by copy number variations (CNVs), which are defined as segments of DNA 1 kb or longer in length than a reference genome^17^, are much more numerous than those influenced by nucleotide substitutions^18^. Gene disruption, dosage alteration, or a change in the level of expression resulted from a significant proportion of CNVs are considered to affect gene function. Several CNVs including loss or gain of the copy in *DNASE1* have been registered in the Database of Genomic Variants (https://dgv.tcag.ca; January 2018). Loss of copy among them could be postulated to cause a reduction in the *in vivo* level of the DNase I activity, thereby affecting the etiology and pathophysiological background of autoimmune conditions such as SLE. However, a genetic characterization of CNVs in *DNASE1* has not been performed.

Comprehensive data on the biochemical-genetic aspects of these genetic variants present in *DNASE1* potentially affecting the *in vivo* DNase I activity would likely be useful for revealing their functionality in genetic predisposition to disease. Therefore, the aim of the present study was to exhaustively evaluate these genetic variants of *DNASEI* that might reduce or abolish the activity of the enzyme, and thus act as potentially pathogenetic variants conferring a genetic predisposition to autoimmunity; we investigated the effect of such genetic variants on the DNase I activity by expression analysis of the DNase I isoform derived from each of them, including missense and nonsense SNPs, and indel (inframe) variants. Furthermore, the distributions of CNVs present in *DNASE1* were examined in German and Japanese populations using a simple quantitative real-time PCR (Q-PCR) method in which the common regions covering all of the CNVs resulting in alterations in the *in vivo* activity levels were chosen as targets for amplification^19^. It was anticipated that our findings would help to clarify the possible role of such genetic variants in genetic disposition to autoimmunity.

## Materials and Methods

### Construction of expression vectors encoding the DNase I isoform derived from a minor allele in each SNP and indel

The nomenclature for SNPs accorded with the recommendations for describing sequence variants (http://www.hgvs.org/mutnomen/examplesDNA.html). As the coding DNA Reference Sequence, the sequence of DNase I (GenBank accession no. AB188151)^20^ was used. Nucleotide and amino acid residues were numbered from the 5′-terminus of the translation initiation codon and the N-terminal amino acid residue of the precursor form of DNase I, respectively.

An expression pcDNA3.1 (+) vector (Invitrogen, San Diego, CA) inserted with the entire coding sequence of human DNase I cDNA derived from the predominant haplotype for genetic variants present in the coding region of the gene was prepared, and used as a wild-type construct. Site-directed mutagenesis using a KOD-Plus Mutagenesis Kit (Toyobo Co., Ltd., Osaka, Japan) with the wild-type construct as a template was employed to separately prepare 70 expression vectors encoding the amino acid-substituted DNase I derived from a minor allele in each missense SNP in *DNASE1*, in the same manner as that for other amino acid-substituted constructs in previous studies^13,14^. Furthermore, expression vectors corresponding to 15 indel (inframe) variants and 7 nonsense SNPs in the gene were separately prepared in the same manner^13,14^. After confirmation of the nucleotide sequence of the insert in each expression vector by sequence analysis, two different clones derived from each vector were purified using the Plasmid Midi kit (Qiagen, Chatsworth, CA, USA) and used for transfection.

### Transient expression of expression vectors and assay for DNase I activity

COS-7 cells were maintained in Dulbecco’s modified Eagle medium containing 1 mM L-glutamine, 50 U/ml penicillin, 50 μg/ml and 10% (v/v) fetal calf serum at 37 °C under 5% CO_2_ in air, and the cells were transiently transfected 4 separate times with 2 μg of each DNase I-related expression vector and 600 ng of pSVβ-galactosidase vector^13^. The cells harvested at 48 hours after transfection were subjected to sonication using a Bioruptor UCD-250 (COSMO BIO Co., LTD, Tokyo, Japan) to prepare lysates for the subsequent assay.

The DNase I activity in the cell lysates was assayed by the single radial enzyme diffusion (SRED) method using a LAS-3000 imaging analyzer (Fuji Film, Tokyo, Japan), as described in our previous report^13^; briefly, each sample was poured into a small cylindrical well punched in an agarose gel plate containing DNA substrate and ethidium bromide. After incubation in a moist chamber for 20 h at 37 °C, the diameter of the circular dark zone derived from digestion of the DNA substrate by DNase I, visible under ultraviolet light, was measured. DNase I activity was determined using a calibration curve constructed by plotting log_10_ DNase I activity against the diffusion radius. The main concept of the SRED method is based on the fact that ethidium bromide fluoresces only with unhydrolyzed DNA and not with DNA that has been digested by DNase I and that the diffusion radius is linearly proportional to the logarithm of DNase I activity. Transfection efficiencies were estimated by cotransfection with a pSV-β-galactosidase vector and subsequent assay of β-D-galactosidase activity in aliquots of cell lysates. After the DNase activity determined by the SRED method in each of the transfections had been separately normalized by the activity of β-D-galactosidase determined in the same transfection, the mean activity of the variant derived from 4 transfections using 2 clones of each construct was expressed relative to that of the wild type; the relative activity being expressed as mean ± standard deviation. Furthermore, in order to examine expression level of each expression vector, expression vectors corresponding to the wild type or variants showing very low DNase I activity were transfected into COS-7 cells as shown above, and DNase I protein present in the cell lysate were detected by Western blot analysis using anti-human recombinant DNase I (Gene Tex, Irvine,CA, USA). As shown in Supplementary Fig. 1, the intensity of the band of each variant was almost similar to that of the wild type, indicating there is no obvious difference on the expression levels between the wild type and each variant.

The activity of each DNase I isoform was compared with that of the wild type using unpaired Student’s *t* test; differences at p < 0.05 were considered to be statistically significant.

### DNA samples from subjects and genotyping of the missense SNPs in *DNASE1* by the PCR-RFLP method

Genomic DNA was extracted using a QIAamp DNA Mini Kit (Qiagen, Chatsworth, CA, USA) from blood samples randomly collected from healthy subjects (*n* = 1,764) derived from 14 populations^13–15^; the Asian population was composed of 110 Japanese (Shimane Prefecture, Japan), 352 Koreans (Busan, South Korea), 193 South Chinese (Shenyang and Guangzhou, China), 112 Mongolians (Ulaanbaatar, Mongolia), 153 Tibetans (Katmandu, Nepal), 35 Sri Lankan Tamils (Kandy, Sri Lanka), 48 Sri Lankan Sinhalese (Kandy, Sri Lanka), and 40 Tamangs (Kotyang, Nepal); the Caucasian population of 136 Turks (Adana area, Southern Turkey), 80 Germans (Munich, Germany) and 199 Mexicans (60 Mestizo, 88 Nahuas, and 51 Huicholes); the African population of 126 Ovambos (Bantusin, Namibia), 105 Ghanaians (Accra, Ghana), and 75 Xhosas (Cape Town, South Africa). Furthermore, 265 Japanese subjects were recruited for the CNV analysis. Written informed consent was obtained from each participant. Also, DNA samples from patients with rheumatoid arthritis (RA) (*n* = 200) were obtained from the immortalized B cell line bank, Japanese Collection of Research Bioresources (Osaka, Japan). The study was approved by the Human Ethics Committees of Shimane University (No. 1024), and the University of Fukui (No. 20180019), and all research was performed in accordance with relevant guidelines and regulations.

Genotyping for each of the SNPs with a minor allele frequency of more than 0.001 revealed in the Ensembl database was separately performed using PCR followed by restriction fragment length polymorphism (RFLP) analysis, according to the previous method^13^. When the substitution site in the SNPs neither suppressed nor created any known restriction enzyme recognition sites, a mismatched PCR-RFLP technique^21^ was employed for this genotyping. The validity of the genotyping results obtained by these methods was directly confirmed by a sequencing analysis of genomic DNAs derived from several representative subjects (*n* = 5) according to the previous studies^13–15^; genomic sequences, including the substitution site of each SNP, were determined by the dideoxy chain-terminating method with the BigDye® Terminator Cycle Sequencing Kit (Applied Biosystems, Foster City, CA, USA) and the sequencing run on a Genetic Analyzer 310 (Applies Biosystems).

### Prediction of the functional effects of amino acid substitutions derived from missense SNPs in *DNASE1* using several prediction tools

In order to predict possible changes in the function of DNase I due to the amino acid substitutions resulting from all the missense SNPs examined, results obtained using Polyphen-2 and SIFT on the variant table of *DNASE1* in the Ensembl database were employed; the prediction outcome of the former can be presented as “benign”, “possibly damaging” or “probably damaging”. Furthermore, PROVEAN v1.1.3 (http://provean.jcvi.org)^22^, SNAP2 (https://rostlab.org/owiki/index.php/Snap2)^23^, and PredictSNP including PANTHER (https://loschmidt.chemi.muni.cz/predictsnp1)^24^ were employed as tools for predicting the functionality of each SNP. Furthermore, the effect of inframe indels on DNase I function was estimated using PROVEAN v1.1.3.

### Estimation of CNV copy number in *DNASE1*

Estimation of CNV copy number in *DNASE1* was performed essentially according to the previous study^19^; we chose the common region of CNVs in *DNASE1* registered in the Database of Genomic Variants (http://dgv.tcag.ca/gb2/gbrowse/dgv2_hg38/), and the single-copy ZNF80 gene (*ZNF80*) as a reference gene^25^. All of the primers used in this study were designed using the NCBI Primer-BLAST software, except those for *ZNF80*, which were selected from the public RTPrimerDB database (RTPrimer DB ID: 1021) as shown in Supplementary Table 1. Each of the gene-specific sequences was PCR-amplified using human genomic DNA as a template and the primer set DN1-S1/-AE or ZNF80-SN/-A1, then cloned into the pCR II vector (Invitrogen), being designated as pCRII/DN1 and pCRII/ZNF, respectively. Next, the chimeric plasmid vector containing both one copy of the DNase gene-specific sequence and the *ZNF80*-specific sequence arranged tandemly was prepared using the PCR gene fusion technique^26^; two PCR amplicons from *DNASE1* and *ZNF80* amplified separately were mixed with the primer set DN1-S1/ZNF-A1 and extended by PCR to form the chimeric sequence. The PCR-amplified product was then cloned into the pCRII vector, and designated as DN1-ZNF. All the constructs were confirmed by DNA sequence analysis and purified using the Plasmid Midi kit (Qiagen) for subsequent analysis.

Q-PCR was performed with the StepOne Plus real-time PCR system (Applied Biosystems)^19,27^. Standard curves for the target CNV region derived from *DNASE1* and *ZNF80* were separately constructed using a 2-fold dilution series of the corresponding chimeric vector preparation as a template. The specificity of the amplified products was evaluated by melting curve analysis. Q-PCR amplification for determination of the CNV copy number was performed in a total volume of 20 μl, containing 10 ng of genomic DNA, 2 pmol of the primer pairs as shown in Supplementary Table 1, and 10 μl of Power SYBR Green Master Mix (Applied Biosystems). The cycling conditions were: 95 °C for 10 min, followed by 40 cycles of 95 °C for 15 s, 61 °C for 1 min. All of the PCR assays were performed in triplicate. The diploid copy number of the CNVs in each DNase gene was calculated using the formula: (amount of *DNASE1* amplicons)/(amount of *ZNF80* amplicons) × 2. The allowable error range for copy number estimation was set at ±0.4, as reported previously^19,27^.

## Results

### Functional effects of the corresponding amino acid substitutions derived from the missense SNPs in *DNASE1*

**Figure 1:**
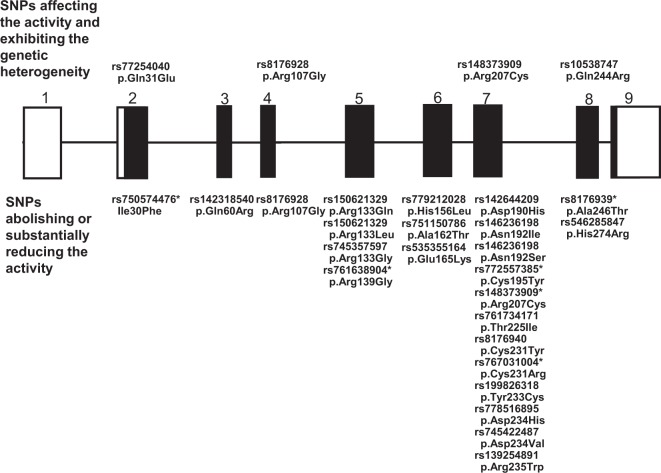
SNPs in *DNASE1* exhibiting genetic heterogeneity, and abolishing or substantially reducing DNase I activity. The DNase I activity in lysates from cells transfected with expression vectors corresponding to these SNPs in *DNASE1* was determined by the SRED method, respectively (Supplementary Table 2). SNPs marked with asterisk correspond to those producing a substantially low-activity form of the enzyme. SNP nomenclature is based on the recommendations for describing sequence variants (http://www.hgvs.org/mutnomen/examplesDNA.htlm). The genomic structure of the human *DNASE1* gene is based upon the NCBI Reference Sequence: NG_009285.1. Exons are shown by solid boxes, in which solid and clear boxes correspond to the translated and untranslated regions of the mRNA, respectively.

We have been examining the effects of amino acid substitutions resulting from a series of missense SNPs located in *DNASE1* on DNase I activity, and previously clarified the functional effects of 61 missense SNPs in *DNASE1*^13–16^. In the present study, in order to evaluate the missense SNPs newly registered in the database, 70 expression vectors producing the amino acid-substituted DNase I protein encoded by the minor allele in each SNP were constructed and transiently expressed in COS-7 cells, and the resulting DNase I activity in the transfected cells was determined by the SRED method. Consequently, in addition to the previous findings^13–16^, we were able to reveal the effect of each amino acid substitution corresponding to a total of 131 missense SNPs in *DNASE1* on the DNase I activity; these SNPs were classified into 4 categories according to their effect on the activity: 57 SNPs not affecting the activity level, 36 reducing it, 20 elevating it, and 18 abolishing it (Supplementary Table 2). Notably, since each activity originating from the amino acid-substituted DNase I corresponding to the latter 18 SNPs could be not detected under our assay conditions, the minor allele in these SNPs was definable as a loss-of-function type resulting in the disappearance of DNase I activity. Since His156, Asp190, Asn192, Asp234 and His274 residues in the DNase I protein are known to be involved in its catalysis^28^, it seems reasonable that all of the 7 missense SNPs involved in these residues would result in loss of function. Furthermore, among the SNPs reducing the activity, the 6 amino acid substitutions resulting from SNPs p.Ile30Phe, p.Phe140Cys, p.Cys195Tyr, p.Arg207Cys, p.Cys231Arg and p.Ala246Thr markedly reduced the activity to less than about 20% of that of the wild type. Thus, we were able to classify the missense SNPs as functional SNPs resulting in loss of function or substantially low activity of DNase I, being distributed mainly in exons 5~7 encoding the posterior half of the DNase I protein (Fig. 1), similarly to DNase 1-like 2 (1L2)^29^.

### *In silico* prediction of the functional effect of missense SNPs in *DNASE1* using several prediction tools

Polyphen-2 available via a Web server has been used as a popular tool for *in silico* prediction of the possible impact of an amino acid substitution derived from missense SNPs on the function of a human protein, and is indispensable for identification of pathogenetic SNPs^30^. Previously, we demonstrated that at least the SNPs causing a loss of function in the DNase 1L2 gene, possible serving as genetic risk factors for the pathogenesis of parakeratosis, could be estimated from Polyphen-2 scores corresponding to “probably damaging” with a relatively high predictive accuracy^29^. However, although predicted to be “probably damaging”, SNPs such as p.Gly127Arg, p.Tyr117Ser and p.Asp129Asn in *DNASE1* were classified as SNPs not affecting the enzyme activity. Since in the present study we determined the functional effects of as many as 131 missense SNPs present in a single gene, *DNASE1*, these data allowed us to substantiate the accuracy of the predicted functional impact of an amino acid substitution corresponding to all of these missense SNP using Polyphen-2, together with the other prediction tools such as SIFT, PROVEAN, PANTHER, SNAP2 and PredictSNP (Supplementary Table 2). The predicted functional effects of these SNPs using the above tools are summarized in Table 1. Any one of the tools predicted that almost all of the 18 SNPs abolishing the activity, and 36 SNPs reducing the activity affected the DNase I activity except for a few that were predicted to be not deleterious would be deleterious, thus confirming their predictive accuracy. On the other hand, among 57 SNPs not affecting the activity and 20 elevating the activity, about half were falsely anticipated to be deleterious by any one of the above tools. These findings demonstrated that at least in *DNASE1* the amino acid substitutions resulting from missense SNPs actually affecting the activity could be relatively well determined to be deleterious, whereas those from SNPs not affecting or elevating the activity were not necessarily non-deleterious. Among the prediction tools evaluated, PredictSNP showed the highest predictive accuracy (Supplementary Table 3). However, among the SNPs predicted to be deleterious, some frequently induced no alteration in the DNase I activity through the corresponding amino acid substitution. Therefore, although prediction tools such as PredictSNP and Polyphen-2 are indispensable for interpretation of genetic variants resulting from missense SNPs, especially the identification of functional SNPs, a combination of functional analysis of genetic variants derived from missense SNPs at the protein level, together with *in silico* analysis, allows actual clarification of their effects on protein functionality.

**Table 1:** Summary^a^ on the prediction of the effect of the amino acid substitution on the DNase I activity resulted from each of the missense SNPs using several prediction tools.

SNPs^b^	Total	Predicted by						
		PolyPhen-2			SIFT		PROVEAN	
		probably damaging	possibly damaging	benign	deleterious	tolerated	deleterious	neutral
Abolishing the activity	18	18	0	0	18	0	18	0
Reducing the activity	36	27	3	6	31	5	32	4
Not affecting the acitivty	57	19	6	32	27	30	25	32
Elevating the acitivy	20	6	3	11	10	10	9	11
		**PANTHER**		**SNAP2**		**PredictSNP**		
		**deleterious**	**neutral**	**effect**	**neutral**	**deleterious**	**neutral**	
Abolishing the activity	18	18	0	17	1	17	1	
Reducing the activity	36	30	6	31	5	29	7	
Not affecting the acitivty	57	24	33	24	33	31	36	
Elevating the acitivy	20	9	11	7	13	6	14	

^a^Total numbers of SNPs categorized according to the prediction results are presented.

^b^The missense SNPs were sorted into the 4 groups based on the effect of the corresponding amino acid substitutions on the DNase I activity.

### Functional effect of nonsense SNPs and indel (inframe) variants in *DNASE1*

**Figure 2:**
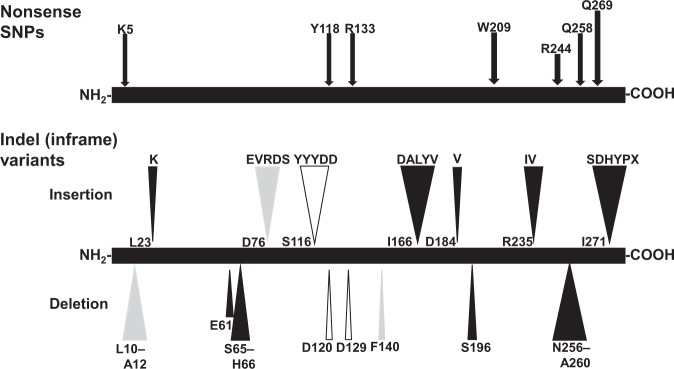
Effect of nonsense and indel (inframe) variants in *DNASE1* on the enzyme activity. The DNase I activity in lysates from cells transfected with expression vectors corresponding to these nonsense and indel (inframe) variants was determined by the SRED method, respectively (Supplementary Table 4). The positions of the amino acid residues affected by mutation are shown on the precursor of the DNase I protein represented as a solid bar. Solid, grey and open arrows indicate the variants abolishing, reducing and not affecting the DNase I activity, respectively.

Although 15 nonsense SNPs and 15 indels (inframe) variants including 7 insertions and 8 deletions in *DNASE1* are registered in the Ensembl database, their effects on DNase I activity have remained unknown. In the present study, we examined the functional effects of 7 nonsense and all 15 indel (inframe) variants (Supplementary Table 4). This revealed that none of the nonsense constructs exhibited DNase I activity, demonstrating that all of the alleles corresponding to those nonsense variants led to a loss of DNase I function. Among them, even the SNP p.Gln269* lacks both the Asp273 and His274 residues in the DNase I protein essential for the catalytic function of the enzyme. Since the other 6 nonsense SNPs lack these essential amino acid residues in the same manner as SNP p.Gln269* (Fig. 2), it is reasonable that cells transfected with these 6 nonsense constructs expressed no DNase I activity. The other 8 nonsense SNPs and 15 variants producing frameshifts which were not examined in the present study are also distributed in *DNASE1*. From these findings, it is plausible to conclude that all of the SNPs producing frameshift or nonsense mutations in *DNASEI* result in loss of function of the enzyme due to the absence of Asp273 and His274 essential for catalysis, and that they can be considered pathogenetic SNPs.

On the other hand, among the 15 indel (inframe) variants, the constructs derived from 5 insertion and 4 deletion SNPs exhibited no DNase I activity in the transfected cells, whereas 3 and 3 SNPs reduced the activity to 20–50% that of the wild type and had no effect on the activity, respectively (Supplementary Table 4). Although the indel (inframe) was predicted by PROVEAN v1.1.3 to have a deleterious effect on DNase I function, variants corresponding to p.delAsp120 and p.delAsp129, and p.Ser116_Tyr117insTyrTyrTyrAspAsp produced levels of enzyme activity similar to that of the wild-type DNase I. Since the sites related to these variants are situated in the loop region on the surface of the DNase I protein (Supplementary Fig. 2)^28^, it is plausible that the insertion of a few amino acid residues or deletion of one residue in the loop region might have little effect on the overall structure of the protein, thus preserving the activity. We have previously demonstrated that hydrophobicity and α-helical structure in the signal sequence region of vertebrate DNase I proteins affect the level of expressed enzyme activity via the post-translational protein targeting pathway^31^. It can be assumed that the deletion corresponding to p.delLeu10_Ala12 would reduce the hydrophobicity and α-helical structure in the signal sequence region, thus diminishing the DNase I activity to about 30% that of the wild type. Also, a 36-bp deletion in exon 2, del46_72, has been identified in Spanish SLE patients^32^, and the corresponding construct exhibited no DNase I activity.

### Genetic heterogeneity of genetic variants in *DNASE1*

Data on the frequency of the second most common allele in the global population defined by the 1000 Genome Project phase 3, designated as the global minor allele frequency (MAF), in *DNASE1* SNPs are available on the Ensembl database (http://asia.ensembl.org/Homo_sapiens/); global MAFs of the variants examined in the present study are shown in Supplementary Tables 2 and 4, and those with a global MAF of more than 0.001 are summarized in Table 2. In the present study, we examined the genetic distribution of all of these *DNASE1* SNPs in the 14 different populations, including three ethnic groups. This revealed that only SNP p.Gln244Arg exhibited genetic polymorphism in all of the populations examined, and heterozygous genotypes for SNPs p.Arg2Ser and p.Gly127Arg, and p.Tyr117Ser were found only in the African and Caucasian populations, respectively, irrespective of no polymorphism level, being consistent with data the 1000 Genome Project phase 3. On the other hand, only one homozygous genotype of the predominant allele, which produces the wild-type DNase I protein, was observed among all of the other SNPs, showing a monoallelic distribution in all of the populations examined. Furthermore, in the RA patients, we examined the distribution of SNPs p.Arg107Gly and p.Arg207Cys abolishing and substantially reducing the activity, respectively, with a global MAF of more than 0.001 and which were present more frequently than other potentially pathogenic SNPs. This revealed that, similarly to the healthy Japanese population in our study, no allele in the SNPs resulting in loss or substantial reduction of the activity was present in the RA patient group.

**Table 2:** All the missense SNPs with global MAF of ≧0.001 in *DNASE1* examined; genetic distribution, activity, effect of the corresponding amino acid substitution on the activity, and prediction of the effect using PolyPhen-2 and PredictSNP.

SNPs	Global MAF^a^	Genetic heterogeneity^b^	Activity^c^	Effect^d^	Prediction by	
					PolyPhen-2	PredictSNP
rs1053874 p.Gln244Arg; c.731 A > G	0.494	polymorphic	0.48 ± 0.015*	reducing	benign	neutral
rs8176927 p.Arg2Ser; c.6 G > T	0.038	only African	1.17 ± 0.17	not affecting	benign	neutral
rs8176919 p.Gly127Arg; c.379 G > A	0.022	only African	2.11 ± 0.52	elevating	damaging	deleterious
rs1799891 p.Pro154Ala; c.460 C > G	0.006	only Japanese^e^	1.43 ± 0.25	not affecting	benign	neutral
rs8176928 p.Arg107Gly; c.319 A > G	0.004	mono-allelic	n.d.	abolishing	probably damaging	deleterious
rs34923865 p.Tyr117Ser; c.350 A > C	0.004	only Caucasian	1.02 ± 0.26	not affecting	probably damaging	deleterious
rs148373909 p.Arg207Cys; c.619 C > T	0.004	only Japanese^e^	0.12 ± 0.022*	reducing	probably damaging	deleterious
rs34907394 p.Glu35Asp; c.105 G > C	0.002	mono-allelic	1.23 ± 0.28	not affecting	benign	neutral
rs144059899 p.Asp129Asn; c.385 G > A	0.002	mono-allelic	1.92 ± 0.81	not affecting	probably damaging	deleterious
rs77254040 p.Gln31Glu; c.91 C > G	0.001	only Japanese^e^	0.43 ± 0.10*	reducing	benign	neutral
rs59621760 p.Asp167Glu; c.501 C > G	0.001	mono-allelic	1.17 ± 0.15	not affecting	possibly damaging	deleterious
rs74892550 p.Val185Ile; c.553 G > A	0.001	mono-allelic	1.49 ± 0.36	not affecting	benign	neutral
rs34186031 p.Pro219Ser; c.655 C > T	0.001	mono-allelic	1.13 ± 0.29	not affecting	benign	neutral

^a^Taken from the Ensembl database (http://asia.ensembl.org/Homo_sapiens/).

^b^Genetic heterogeneity for each of SNP in our study populations are shown.

^c^The values are expressed as relative activity of each amino acid-substituted construct in the cell lyzates to that of the wild-type, representing the mean ± SD (*n* = 4); n.d., the activity derived from the corresponding amino acid substituted construct could not be detected under our assay conditions. The asterisks show the activity significantly reduced compared to that of the wild type.

^d^The activity of each DNase I isoform was compared with that of the wild type using unpaired Student’s *t* test; differences at p < 0.05 were considered to be statistically significant. Based upon the significant effect on the activity, SNPs could be classified into 4 categories.

^e^The corresponding minor alleles of each SNP were found in the other Japanese population recruited in the previous studies^13^.

On the basis of these findings, it was possible to select SNPs not only showing genetic heterogeneity but also affecting the enzyme activity (Fig. 1). It is worth noting that SNP p.Gln244Arg, which results in a reduction of enzyme activity to about half that of the wild type, is distributed worldwide at the polymorphic level, whereas SNPs p.Arg107Gly abolishing the activity, and p.Arg207Cys substantially reducing the activity are found only in African populations at the polymorphic level, and worldwide irrespective of no polymorphism level, respectively.

### Low genetic heterogeneity of CNVs in *DNASE1*

**Figure 3:**
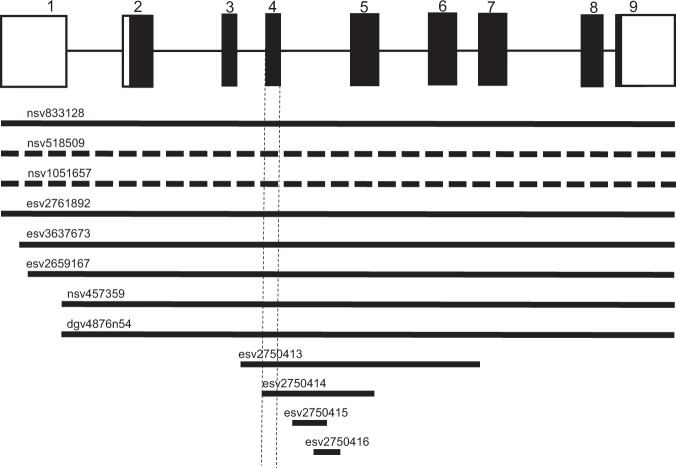
The CNVs in *DNASE1*. The genomic structure of the human DNase I gene is based upon the NCBI Reference Sequence: NG_009285.1. All the CNVs in the gene registered in the Database of Genomic Variants (http://dgv.tcag.ca/gb2/gbrowse/dgv2_hg38/) are shown. The solid and dashed bars indicate loss and gain of copy, respectively, in the CNVs. The region of the gene shown by the dashed lines is the target region used for Q-PCR analysis of each gene.

Several CNVs have been found in *DNASE1*, especially those resulting in loss of copy that would be expected to markedly reduce the *in vivo* level of DNase I activity. In the present study, we focused on the target region common to all of the CNVs for *DNASE1* likely giving rise to alterations in the *in vivo* levels of enzyme activity through loss or gain of copy in the CNVs, thus clarifying the overall effects of the CNVs on *in vivo* DNase I activity; for amplification by Q-PCR we selected exon 4 in *DNASE1* as the common target region, which covers all of the CNVs (Fig. 3). Standard curves for the target CNV region, together with the single-copy ZNF80 gene as a reference, were generated using a 2-fold serially diluted series of each chimeric vector as a template (Supplementary Fig. 3). For *DNASE1* CNV analysis, the efficiency of PCR for the reference gene (*ZNF80*) and the target CNV was 95.8 ± 3.26% (slope coefficient, −3.43 ± 0.0865) and 98.1 ± 3.74% (slope coefficient, −3.37 ± 0.0916), respectively. Since the quantitative accuracy of Q-PCR analysis depends on proper normalization, these results demonstrated that copy number estimation using this method was reliable. Furthermore, simulated analysis of the *DNASE1* CNV copy number in which mixtures of three vectors – pCRII/DN1, pCRII/ZNF or DN1-ZNF – in varying proportions were used as a template, yielded the expected number of copies, indicating the reliability of this method. Therefore, this simple, newly developed Q-PCR method for CNV analysis permits reliable copy number determination for CNVs located in *DNASE1*.

Next, we examined the distribution of the *DNASE1* CNVs in Japanese and German populations. To our knowledge, this is the first study to have comprehensively clarified the distribution of the CNVs in *DNASE1*. However, the present CNV analysis demonstrated that only the 2 diploid copy number was present in the CNVs of both populations (Table 3). Notably, it was clarified that loss of the copy giving rise to abrogation of the enzyme activity was not widely distributed. These findings allow us to conclude that *DNASE1* shows remarkably low genetic diversity, in terms of CNVs that alter the *in vivo* DNase activity. Based on our expression analysis of the recombinant forms of DNase I shown above, 18 missense, 7 nonsense and 8 indel (inframe) SNPs producing loss-of-function variants of the enzyme encoded by *DNASE1* have been identified. However, all of these functional variants resulting in loss of function, except for SNP p.Arg107Gly with a MAF of 0.004, exhibit a remarkably low distribution (Table 2), similar to that of the CNVs. Thus, it is plausible to assume that *DNASE1* has been well conserved at the activity level during the evolution of human populations, thereby avoiding any marked reduction of the activity through a loss of such functional variants, as already demonstrated for other members of the human DNase family: DNase I-like 1 (1L1)^33^, DNase 1L2^29,34^, DNase 1L3^14,16^ and DNase II^35^.

**Table 3:** Estimated diploid copy number^a^ of the CNVs in *DNASE1* in Japanese and German populations.

Population	Estimated copy number					Copy number calculated (mean ± SD)			
	1	2	3	4	total	1	2	3	4
Japanese	0	265	0	0	265	NA	1.82 ± 0.13	NA	NA
German	0	80	0	0	80	NA	1.88 ± 0.12	NA	NA

^a^The diploid copy numbers of the CNVs in *DNASE1* were estimated based on the copy number calculated using the following formula: (amount of *DNASE1* amplicons)/(amount of *ZNF80* amplicons) × 2.

NA: not applicable.

## Discussion

Napirei *et al*.^8^ demonstrated that knockout of *DNASE1* caused mice to develop SLE-like syndromes. In Japanese and German patients with autoimmune disease, novel nonsense (p. Lys5*) and missense (p.Val111Met) variants of *DNASE1*, respectively, have been identified^9,10^, the latter reducing DNase I activity to about one fourth of that of the wild type (Supplementary Table 2). Also, the 46_72*del* mutation in exon 2 of the gene was found in Spanish SLE patients^32^, and our expression analysis showed the corresponding deletion to lead to a loss of function. Patients with SLE are known to have levels of serum DNase I activity lower than those in healthy subjects^36–39^. Furthermore, serum DNase I activity is positively correlated with the SLE disease activity index-2K^40^. In lupus nephritis, which is the most serious complication of SLE, down-regulation of renal DNase I has been demonstrated to result in reduced chromatin fragmentation and deposition of extracellular chromatin-IgG complexes in the glomerular basement membranes in individuals producing IgG anti-chromatin antibodies^41,42^. Furthermore, it has been postulated that insufficient degradation of NETs by DNase I would allow NETs to persist, thus permitting presentation of chromatin-associated self-antigens, and thereby promoting SLE^2^. Therefore, genetic variants in *DNASE1* producing DNase I isoforms with reduced and/or abolished activity may form the basis of genetic predisposition to abnormally low *in vivo* DNase I activity, thereby leading to autoimmune dysfunction. However, there is a lack of comprehensive information on genetic variants in *DNASE1* that might potentially affect enzyme activity. Combined with our previous data^13–16^, the present exhaustive examination of the functional effects of a series of genetic variants of *DNASE1*, including missense and nonsense SNPs, and indel (inframe) variants, on the activity of the enzyme, is the first to have comprehensively clarified the functionality of such genetic variants. We were able to identify 18 and 6 missense SNPs resulting in loss and marked reduction, respectively, of the enzyme function (Fig. 1). Furthermore, we demonstrated that 7 nonsense SNPs and 9 indel (inframe) variants abolished the DNase I activity (Fig. 2), allowing us to assume that all of the 8 other nonsense and 15 frameshift SNPs would likely exert a pathogenetic effect through loss of the activity. Accordingly, a total of 60 genetic variants in the gene inducing abrogation or marked reduction of the DNase I activity, irrespective of their low distribution in the populations, were identified as conferring a genetic risk of autoimmunity. Therefore, it is plausible that individuals who are homozygous or show compound heterozygosity for each of the minor alleles of these 60 pathogenetic variants will likely have lower levels of *in vivo* DNase I activity than individuals with other genotypes, thus being at potential risk of developing autoimmune dysfunction such as SLE. In this study, since the SNPs p.Arg107Gly and p.Arg207Cys abolishing and substantially reducing the activity, respectively, were distributed more frequently than other potentially pathogenic SNPs (Table 2), we examined their distributions in patients with RA as a representative autoimmune disease, using DNA samples obtained from a publicly accessible DNA bank. However, no allele in the SNPs resulting in loss or substantial reduction of the activity was found in the RA patients. In order to clarify any clinical associations of these 60 pathogenetic variants with the incidence of autoimmune disease, further studies will need to examine the correlation between the *in vivo* levels of DNase activity and the prevalence of autoimmune diseases for each genetic *DNASE1* variant in larger groups of patients with autoimmune disease.

Only the worldwide polymorphism SNP rs1053874 (p.Gln244Arg; c.731 A > G) in *DNASE1* has been reported to be associated with SLE susceptibility^43,44^. The amino acid substitution of Arg for Gln at position 244 of the DNase I protein resulting from this SNP appears to reduce the specific activity of the enzyme to about half that of the wild-type protein harboring Gln244 (Table 2). In patients with SLE, Shin *et al*.^11^ demonstrated that the frequency of the homozygote for the *G* allele corresponding to Arg244 was much higher in individuals possessing the autoantibodies than in patients who did not, being partly attributable to production of the low activity-harboring DNase I isoform by the *G* allele in SNP p.Gln244Arg. The presence of a 56-bp variable-number tandem repeat polymorphism (VNTR) with 6 types of different repeats in intron 4 of *DNASE1* in linkage disequilibrium with SNP p.Gln244Arg was revealed^45^. Among these alleles, SLE patients showed a higher distribution of allele 5 than the controls^46,47^. The fact that allele 5, together with allele 4, in the VNTR is associated with the *G* allele in SNP p.Gln244Arg, producing a DNase I isoform with low activity, suggests that the VNTR in *DNASE1* likely confers susceptibility to SLE. On the other hand, in a study of Tunisian patients, no significant association of SNP rs8176927 (p.Arg2Ser; c.6 G > T) with SLE, rheumatoid arthritis or Sjögren syndrome was found^48^. Since the amino acid substitution resulting from the SNP does not affect the DNase I activity, despite its slightly frequent distribution in various populations (Table 2), the lack of such an association may not be surprising.

Recently, it has been noted that NETs formed in the process of NETosis may play a role in non-infectious diseases such as autoimmunity, atherosclerosis, vasculitis, and thrombosis^49,50^. With regard to MI, Mangold *et al*.^3^ have demonstrated that extracellular DNase can serve as an endogenous regulator of NTEs, DNase activity at the lesion site showing a negative correlation with the coronary NET burden, infarct size, and area at risk. Furthermore, administration of DNase I to mice with acute MI/reperfusion injury significantly reduced infarct size and the plasma levels of nucleosomes, thereby improving cardiac function^51^. Impaired DNase I-mediated degradation of NETs is also reported to be associated with acute thrombotic microangiopathies^4^. These clinical findings suggest that reduction of *in vivo* DNase I levels linked to genetic factors may have a potential role in the pathogenesis of such diseases. In fact, we have demonstrated that the allele producing a low-activity DNase I isoform in common SNP p.Gln244Arg was associated with a higher incidence of MI^12^. Previous studies have demonstrated that the genes encoding members of the human DNase family, DNases 1L1, 1L2, 1L3 and II^14,16,33–35^, exhibit remarkably low genetic heterogeneity with regard to missense SNPs, irrespective of whether or not they affect the respective DNase activity. In contrast, SNPp.Gln244Arg in *DNASE1* has a marked effect on the enzyme activity and is distributed worldwide at the polymorphic level. These findings suggest that DNase I may be implicated in several diseases as a genetic background factor through reduction of its activity by this polymorphic SNP. Therefore, it seems likely that only *DNASE1* SNPp.Gln244Arg, which reduces the activity of the enzyme and has a worldwide distribution, could be a genetic background factor conferring predisposition to at least SLE and MI.

## Supplementary information

Supplementary Information

Supplementary table 2

Supplementary table 4
