# Supplementary material for: Evaluation of the functional effects of genetic variants‒missense and nonsense SNPs, indels and copy number variations‒in the gene encoding human deoxyribonuclease I potentially implicated in autoimmunity
Source: Sci Rep. 2019 Sep 20;9:13660. doi: 10.1038/s41598-019-49935-y (PMC6754452; doi:10.1038/s41598-019-49935-y)
Supplement: Supplementary file 1 — Supplementary Information [file 41598_2019_49935_MOESM1_ESM.pdf]

Evaluation of the functional effects of genetic variants–missense and nonsense SNPs, indels and copy number variations–in the gene encoding human deoxyribonuclease I potentially implicated in autoimmunity

Misuzu Ueki[1], Kaori Kimura-Kataoka[2], Junko Fujihara[2], Reiko Iida[3], Yasuyuki Kawai[4], Akari Kusaka[2], Takamitsu Sasaki[2], Haruo Takeshita[2*], Toshihiro Yasuda[1]

[1]Department of Medical Genetics and Biochemistry, Faculty of Medical Sciences, University of Fukui, Eiheiji, Fukui, Japan
[2]Department of Legal Medicine, Shimane University School of Medicine, Enya, Izumo, Japan
[3]Department of Life Sciences, Faculty of Medical Sciences, University of Fukui, Eiheiji, Fukui, Japan
[4] Department of Cardiology, Kanazawa Medical University, Uchinada, Ishikawa, Japan

*Corresponding author
E-mail: htakeshi@med.shimane-u.ac.jp (HT)

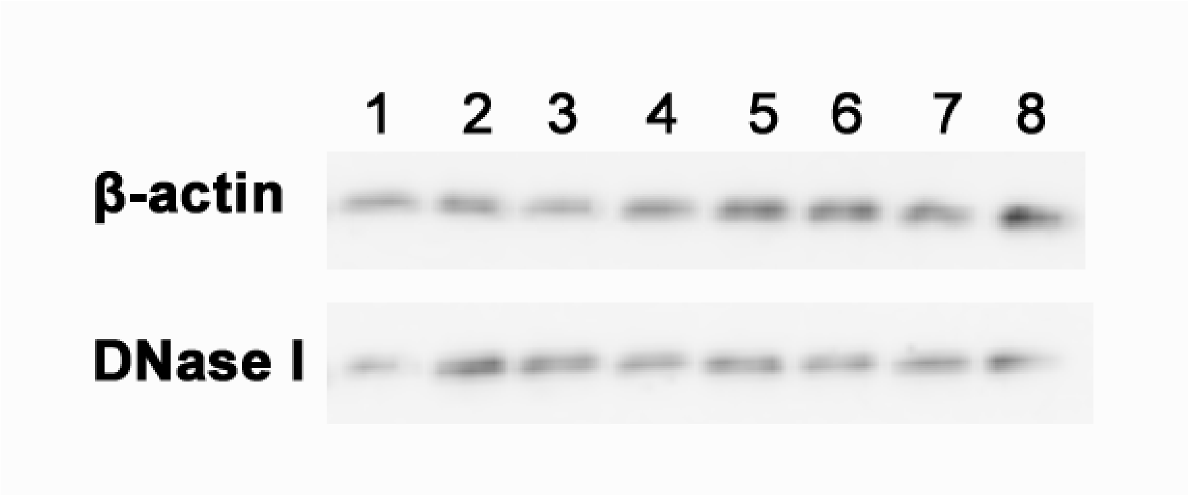

Supplementary figure 1. Western blot analysis of DNase I variants expressed in COS-7 cells.

DNase I protein present in the cell lysate transfected with expression vectors corresponding to the wild type or seven representative variants was detected by Western blot analysis using anti-human DNase I antibody. Expression of β-actin is shown as an internal control. Lane 1, p.Tyr85Cys; lane 2, p.Asn78His; lane 3, p.Ile30Phe; lane 4, wild type; lane 5, p.Arg139Gly; lane 6, p.Cys195Tyr; lane 7, p.Cys231Arg; lane 8, p.Gly240Arg.

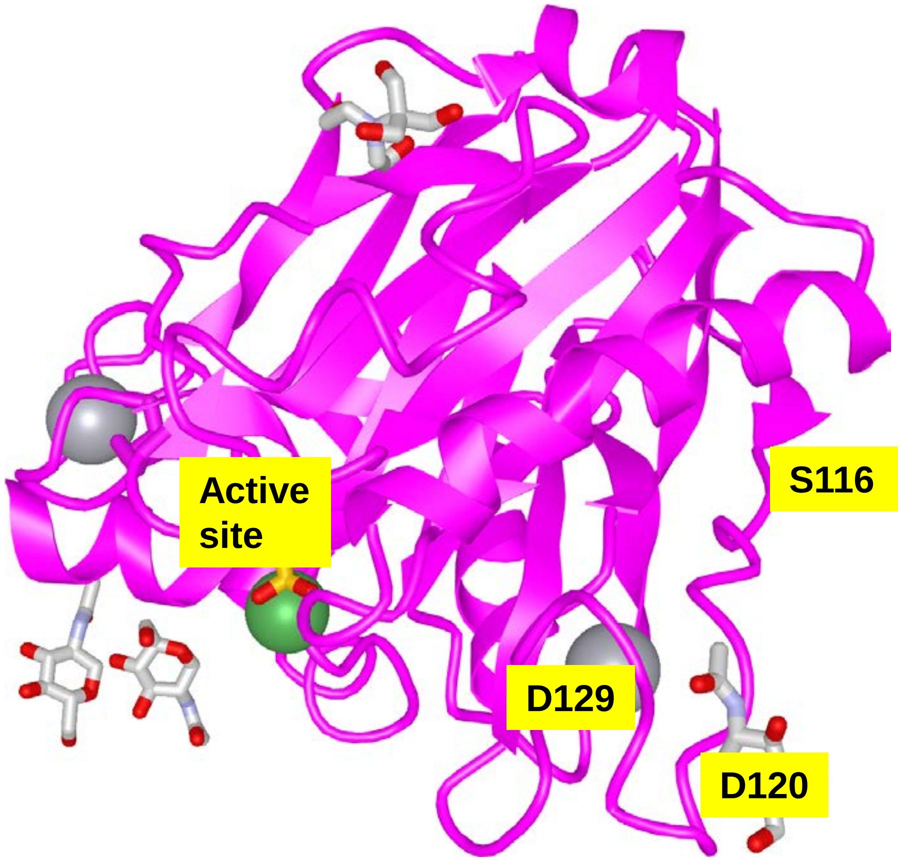

Supplementary figure 2. The 3-D structure of human DNase I protein

The 3-D structure of human DNase I is taken from Database (https://www.ncbi.nlm.nih.gov/Structure/). Each site on the DNase I protein corresponding to Indels p.delAsp120 and p.delAsp129, and p.Ser116_Tyr117insTyrTyrTyrAspAsp are shown.

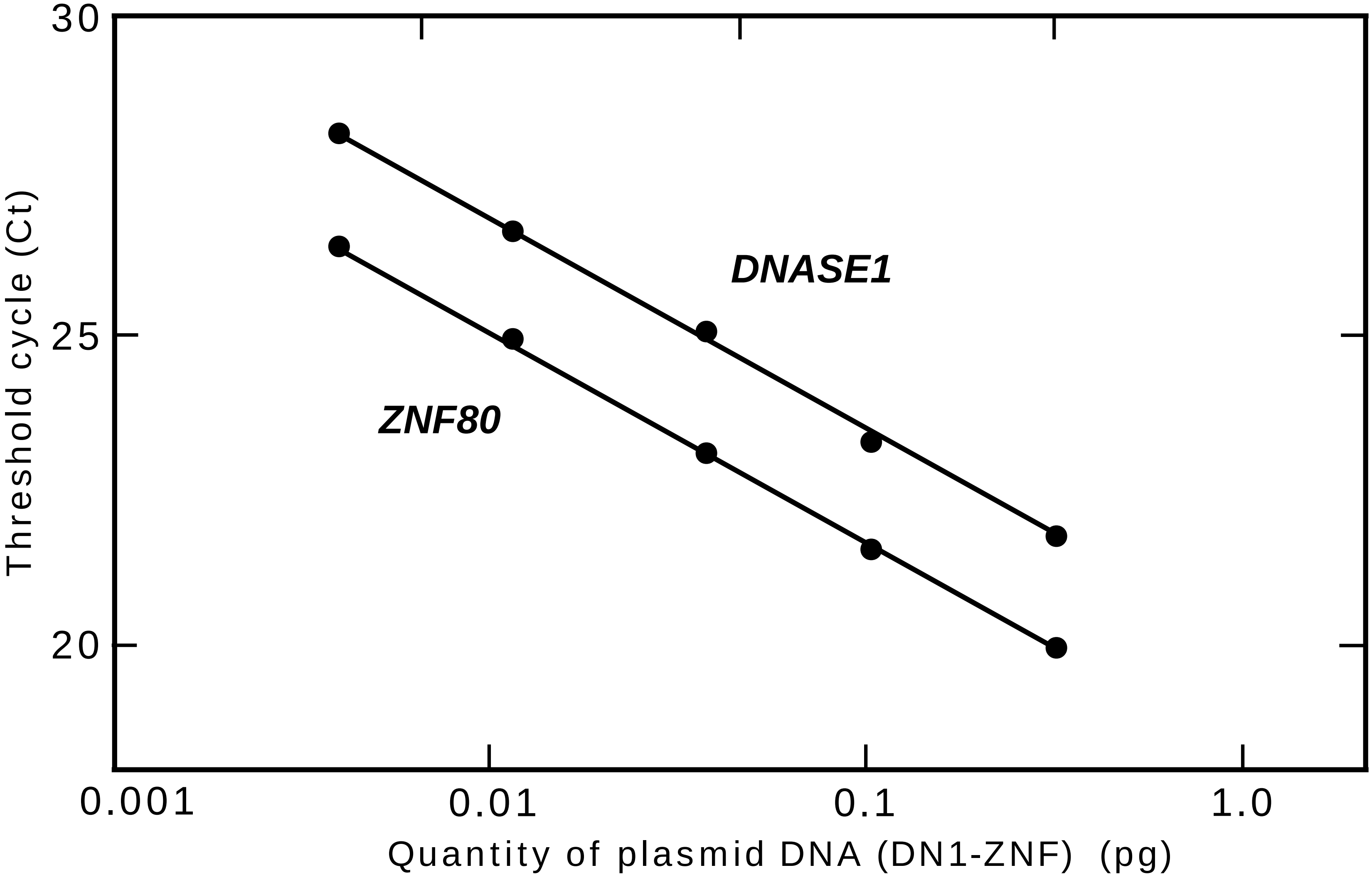

Supplementary figure 3. Standard curve of Q-PCR used for copy number analyses of the *DNASE1* CNV.

The standard curves were constructed by plotting a known amount of the chimeric vector, DN1-ZNF, in serial 2-fold dilutions (0.014 to 0.23 pg) against the corresponding threshold cycles (Ct values) of the amplification plots.

Supplementary table 1. **Primers used for the *DNASE1* CNV analysis**

Gene	Primer[a)]	Orientation	Sequence (5’-3’)[b)]	Positions[c)]	Amplicon size (bp)
DNASE1	DN1-S1	forward	GGAGCGCTACCTGTTCGTGT	8224–8243	89
	DN1-AE	reverse	AGCTGCAGGTCACAGCTTTC-<br>CTTCCCTGCTCTGTTGGGGC		
	DN1-A1	reverse	CTTCCCTGCTCTGTTGGGGC	8293–8313	
ZNF80	ZNF80-S1	forward	CTGTGACCTGCAGCTCATCCT	1431–1452	120
	ZNF80-SN	forward	GAAAGCTGTGACCTGCAGCTCATCCT		
	ZNF80-A1	reverse	TAAGTTCTCTGACGTTGACTGATGTG-<br>ATGTG	1526–1551	

a) For the Q-PCR analysis for *DNASE1* and *ZNF80*, primer sets DN1-S1/-A1 or ZNF80-S1/-A1 were used, respectively.

b) Nucleotide tags for construction of a chimeric plasmid DNA are underlined.

c) The positions of each primer are based on the genomic sequences of each genes; DNase I, NCBI Reference Sequence: NG_009285.1; ZNF80, NCBI Reference Sequence: NC_000003.12.

Supplementary table 3. **Evaluation of the tools used for prediction of the functional effect of the amino acid substitution derived from the 131 missense SNPs in *DNASE1* examined in the present study**

Prediction tool	Sensivity	Specificity	Positive predictive value	Negative predictive value	Accuracy
PolyPhen-2[b)]	0.889	0.558	0.585	0.878	0.695
SIFT	0.907	0.519	0.570	0.889	0.679
PROVEAN	0.926	0.558	0.595	0.915	0.710
PANTHER	0.889	0.571	0.593	0.880	0.702
SNAP2	0.889	0.597	0.608	0.885	0.718
PredictSNP	0.852	0.704	0.687	0.862	0.733

a) SNPs abolishing and reducing the activity, and ones not affecting and elevating the activity were combined as ones damaging the DNase I function, and ones not damaging the DNase I function, respectively.

b) SNPs predicted to be “probably damaging” and “possibly damaging” were combined as ones predicted to be “damaging”.
