# Supplementary material for: Evaluation of the functional effects of genetic variants‒missense and nonsense SNPs, indels and copy number variations‒in the gene encoding human deoxyribonuclease I potentially implicated in autoimmunity
Source: Sci Rep. 2019 Sep 20;9:13660. doi: 10.1038/s41598-019-49935-y (PMC6754452; doi:10.1038/s41598-019-49935-y)
Supplement: Supplementary file 2 — Supplementary table 2 [file 41598_2019_49935_MOESM2_ESM.pdf]

Evaluation of the functional effects of genetic variants–missense and nonsense SNPs, indels and copy number variations–in the gene encoding human deoxyribonuclease I potentially implicated in autoimmunity

Misuzu Ueki[1], Kaori Kimura-Kataoka[2], Junko Fujihara[2], Reiko Iida[3], Yasuyuki Kawai[4], Akari Kusaka[2], Takamitsu Sasaki[2], Haruo Takeshita[2*], Toshihiro Yasuda[1]

[1]Department of Medical Genetics and Biochemistry, Faculty of Medical Sciences, University of Fukui, Eiheiji, Fukui, Japan
[2]Department of Legal Medicine, Shimane University School of Medicine, Enya, Izumo, Japan
[3]Department of Life Sciences, Faculty of Medical Sciences, University of Fukui, Eiheiji, Fukui, Japan
[4] Department of Cardiology, Kanazawa Medical University, Uchinada, Ishikawa, Japan

*Corresponding author
E-mail: htakeshi@med.shimane-u.ac.jp (HT)

Supplementary table 2. **Summary on all the 131 missense SNPs in DNASE1 classified into 4 categories (ones not affecting the activity, abolishing the activity, reducing the activity or elevating the activity) [a] including our previous studies [12–16]; the activity of the amino acid substituted DNase I, global MAF and prediction on the effect of the amino acid substitution on the activity using several prediction tools.**

SNP	Activity[b]	Global MAF[c]	Prediction by					
			Polyphen-2[d]	SIFT	PROVEAN	PANTHER	SNAP2	PredictSNP
**SNPs not affecting the activity**								
rs8176927 p.Arg2Ser; c.6G>T	1.17±0.17	0.038	benign	tolerated	neutral	neutral	neutral	neutral
rs200484716 p.Gly3Ser; c.8G>A	1.17±0.17	<0.001	benign	tolerated	neutral	neutral	neutral	neutral
rs148015097 p.Ala14Val; c.41C>T	1.10±0.35	(-)	benign	tolerated	neutral	neutral	neutral	neutral
rs201161491 p.Ile25Met; c.75C>G	1.12±0.21	<0.001	benign	deleterious	neutral	neutral	neutral	deleterious
rs141673463 p.Ala26Thr; c.76G>A	1.14±0.50	(-)	possibly d.	tolerated	neutral	neutral	effect	deleterious
rs562520867 p.Phe28Val; c.82T>G	0.89±0.24	<0.001	probably d.	deleterious	deleterious	deleterious	effect	deleterious
rs34907394 p.Glu35Asp; c.105G>C	1.23±0.28	0.002	benign	tolerated	neutral	neutral	neutral	neutral
rs551458320 p.Glu35Ala; c.104A>C	0.78±0.019	<0.001	benign	deleterious	deleterious	neutral	neutral	neutral

rs369619441 p.Met38Thr; c.113T>C	1.43±0.41	(-)	possibly d.	tolerated	deleterious	neutral	neutral	neutral	
rs756483303 p.Ser39Phe; c.116C >T	0.98±0.10	(-)	probably d.	deleterious	deleterious	deleterious	effect	deleterious	
rs745479565 p.Asn40Asp; c.118A>G	0.81±0.14	(-)	probably d.	tolerated	deleterious	deleterious	neutral	neutral	
rs370054136 p.Val44Ile; c.130G>A	1.32±0.10	<0.001	benign	tolerated	neutral	neutral	neutral	neutral	
rs775904555 p.Ser65Ile; c.194G>T	0.91±0.14	(-)	benign	deleterious	deleterious	deleterious	neutral	neutral	
rs538284922 p.His66Tyr; c.196C>T	0.71±0.19	<0.001	benign	tolerated	deleterious	deleterious	neutral	neutral	
rs571444570 p.Val70Met; c.208G>A	1.24±0.028	<0.001	probably d.	deleterious	deleterious	deleterious	neutral	neutral	
rs534046382 p.Asp75Tyr; c.223G >T	0.92±0.19	<0.001	probably d.	deleterious	deleterious	deleterious	effect	deleterious	
rs140530129 p.Ala81Thr; c.241G>A	0.95±0.074	<0.001	benign	tolerated	neutral	neutral	neutral	neutral	
rs563628785 p.Pro82Ser; c.244C>T	0.88±0.11	<0.001	benign	tolerated	deleterious	neutral	neutral	neutral	

rs190768401<br>p.Arg95Gln; c.284G>A	1.14±0.39	(-)	probably d.	deleterious	deleterious	deleterious	effect	deleterious	
rs772385567<br>p.Arg101His; c.302G>A	0.71±0.19	(-)	probably d.	deleterious	deleterious	deleterious	effect	deleterious	
rs199986334<br>p.Ala113Val; c.338C>T	1.36±0.42	(-)	benign	tolerated	neutral	neutral	neutral	neutral	
rs530214101<br>p.Val114Met; c.340G>A	1.06±0.21	<0.001	benign	tolerated	neutral	neutral	neutral	neutral	
rs34923865<br>p.Tyr117Ser ;c. 350A>C	1.02±0.26	0.004	probably d.	deleterious	deleterious	deleterious	effect	deleterious	
rs990252602<br>p.Tyr119Cys; c.356A>G	1.29±0.37	(-)	probably d.	deleterious	deleterious	deleterious	effect	deleterious	
rs762047836<br>p.Asp120Val; c.359A>T	1.36±0.42	(-)	probably d.	deleterious	deleterious	deleterious	effect	neutral	
rs144059899<br>p.Asp129Asn; c.385G>A	1.92±0.81	0.002	probably d.	deleterious	deleterious	deleterious	effect	deleterious	
rs76397583<br>p.Asn132Ser; c.395A>G	1.45±0.66	(-)	benign	tolerated	neutral	neutral	neutral	neutral	
rs768394464<br>p.Glu134Asp; c.402G>C	0.94±0.13	(-)	probably d.	deleterious	deleterious	deleterious	effect	neutral	
rs374554105	1.42±0.27	(-)	benign	deleterious	neutral	deleterious	effect	deleterious	

p.Arg143Trp; c.427C>T								
rs755563219<br>p.Glu146Lys; c.436G>A	0.86±0.13	(-)	benign	tolerated	neutral	neutral	neutral	neutral
rs1799891<br>p.Pro154Ala; c.460C>G	1.43±0.25	0.006	benign	deleterious	deleterious	neutral	effect	neutral
rs552883126<br>p.Gly160Glu; c.479G>A	0.74±0.20	(-)	benign	tolerated	neutral	neutral	neutral	neutral
rs566411600<br>p.Asp161Gly; c.482A>G	1.00±0.14	(-)	benign	tolerated	deleterious	neutral	effect	neutral
rs59621760<br>p.Asp167Glu; c.501C>G	1.17±0.15	0.001	possibly d.	deleterious	deleterious	neutral	effect	deleterious
rs140745748<br>p.Val172Ile; c.514G>A	1.21±0.71	(-)	probably d.	deleterious	neutral	deleterious	effect	deleterious
rs761406088<br>p.Asp184Ala; c.551A>C	1.18±0.04	(-)	probably d.	deleterious	deleterious	neutral	effect	deleterious
rs74892550<br>p.Val185Ile; c.553G>A	1.49±0.36	0.001	benign	tolerated	neutral	neutral	neutral	neutral
rs147093089<br>p.Met186Val; c.556A>G	0.78±0.41	(-)	benign	tolerated	neutral	neutral	neutral	neutral
rs747813186<br>p.Pro200Ser; c.598C>T	1.14±0.076	(-)	benign	tolerated	neutral	neutral	neutral	neutral

rs769377487 p.Ser201Phe; c.602C>T	0.91±0.15	(-)	probably d.	deleterious	deleterious	deleterious	effect	deleterious
rs752868705 p.Arg207His; c620G>A	1.18±0.10	(-)	probably d.	deleterious	deleterious	deleterious	effect	deleterious
rs750946482 p.Pro212Ser; c.634C>T	1.36±0.38	(-)	benign	tolerated	neutral	neutral	neutral	neutral
rs34186031 p.Pro219Ser; c.655C>T	1.13±0.29	0.001	benign	tolerated	deleterious	deleterious	neutral	neutral
rs146249371 p.Ala232Gly; c.695C>G	1.29±0.12	(-)	benign	deleterious	deleterious	neutral	neutral	deleterious
rs375616517 p.Arg235Lys; c.704G>A	1.13±0.11	(-)	probably d.	deleterious	deleterious	deleterious	effect	deleterious
rs200149984 p.Val238Leu; c.712G>C	0.95±0.37	<0.001	benign	tolerated	neutral	neutral	neutral	neutral
rs757847926 p.Met241Ile; c.723G>A	0.84±0.076	(-)	benign	tolerated	neutral	neutral	neutral	neutral
rs747959505 p.Gly245Arg; c.733G>C	1.19±0.32	(-)	benign	tolerated	neutral	neutral	neutral	neutral
rs148684969 p.Val247Ile; c.739G>A	1.05±0.077	<0.001	benign	tolerated	neutral	neutral	neutral	neutral

rs750030744<br>p.Asp250Asn; c.748G>A	0.86±0.23	(-)	benign	tolerated	neutral	neutral	neutral	neutral
rs200538894<br>p.Ser251Leu; c.752C>T	1.29±0.30	(-)	probably d.	deleterious	deleterious	deleterious	effect	deleterious
rs553553015<br>p.Ala252Val; c.755C>T	1.48±0.36	(-)	possibly d.	deleterious	deleterious	deleterious	effect	neutral
rs777733131<br>p.Pro254Arg; c.761C>G	1.20±0.32	(-)	possibly d.	deleterious	deleterious	neutral	effect	neutral
rs142079857<br>p.Ala259Gly; c.776C>G	1.39±0.19	<0.001	benign	tolerated	neutral	neutral	neutral	neutral
rs201413861<br>p.Ala260Gly; c.779C>G	1.36±0.24	<0.001	possibly d.	tolerated	deleterious	neutral	neutral	neutral
rs368045366<br>p.Tyr261Cys; c.782A>G	0.92±0.20	(-)	probably d.	deleterious	deleterious	deleterious	effect	deleterious
rs8176924<br>p.Gly262Asp; c.785G>A	1.16±0.54	(-)	benign	tolerated	deleterious	neutral	neutral	neutral
**SNPs abolishing the activity**								
rs142318540<br>p.Gln60Arg; c.179A>G	n.d.	<0.001	probably d.	deleterious	deleterious	deleterious	effect	deleterious
rs8176928<br>p.Arg107Gly; c.319A>G	n.d.	0.004	probably d.	deleterious	deleterious	deleterious	effect	deleterious

rs150621329<br>p.Arg133Gln; c.398G>A	n.d.	<0.001	probably d.	deleterious	deleterious	deleterious	effect	deleterious
rs150621329<br>p.Arg133Leu; c.398G>T	n.d.	(-)	probably d.	deleterious	deleterious	deleterious	effect	deleterious
rs745357597<br>p.Arg133Gly; c.397C>G	n.d.	(-)	probably d.	deleterious	deleterious	deleterious	effect	deleterious
rs779212028<br>p.His156Leu; c.467A>T	n.d.	(-)	probably d.	deleterious	deleterious	deleterious	effect	deleterious
rs751150786<br>p.Ala162Thr; c.484G>A	n.d.	(-)	probably d.	deleterious	deleterious	deleterious	neutral	neutral
rs535355164<br>p.Glu165Lys; c.493G>A	n.d.	<0.001	probably d.	deleterious	deleterious	deleterious	effect	deleterious
rs142644209<br>p.Asp190His; c.568G>C	n.d.	(-)	probably d.	deleterious	deleterious	deleterious	effect	deleterious
rs146236198<br>p.Asn192Ile; c.575A>T	n.d.	<0.001	probably d.	deleterious	deleterious	deleterious	effect	deleterious
rs146236198<br>p.Asn192Ser; c.575A>G	n.d.	(-)	probably d.	deleterious	deleterious	deleterious	effect	deleterious
rs761734171<br>p.Thr225Ile; c.674C>T	n.d.	(-)	probably d.	deleterious	deleterious	deleterious	effect	deleterious
rs8176940	n.d.	(-)	probably d.	deleterious	deleterious	deleterious	effect	deleterious

p.Cys231Tyr; c.692G>A								
rs199826318 p.Tyr233Cys; c.698A>G	n.d.	<0.001	probably d.	deleterious	deleterious	deleterious	effect	deleterious
rs778516895 p.Asp234His; c.700G>C	n.d.	(-)	probably d.	deleterious	deleterious	deleterious	effect	deleterious
rs745422487 p.Asp234Val; c.701A>T	n.d.	(-)	probably d.	deleterious	deleterious	deleterious	effect	deleterious
rs139254891 p.Arg235Trp; c.703A>T	n.d.	(-)	probably d.	deleterious	deleterious	deleterious	effect	deleterious
rs546285847 p.His274Arg; c.821A>G	n.d.	<0.001	probably d.	deleterious	deleterious	deleterious	effect	deleterious
**SNPs reducing the activity**								
rs750574476 p.Ile30Phe; c.88A>T	0.16±0.010	(-)	probably d.	deleterious	deleterious	deleterious	effect	deleterious
rs776458509 p.Ile30Thr; c.89T>C	0.340±0.002	(-)	probably d.	deleterious	deleterious	deleterious	effect	deleterious
rs77254040 p.Gln31Glu; c.91C>G	0.43±0.10	0.001	benign	deleterious	neutral	neutral	effect	neutral
rs755123150 p.Phe33Ser; c.98T>C	0.27±0.045	(-)	probably d.	deleterious	deleterious	deleterious	effect	deleterious
rs769521038	0.35±0.040	(-)	probably d.	deleterious	deleterious	deleterious	effect	deleterious

p.Ile47Thr; c.140T>C								
rs143865851 p.Arg53Cys; c.157C>T	0.20±0.050	<0.001	possibly d.	deleterious	deleterious	deleterious	neutral	deleterious
rs144227093 p.Tyr54Cys; c.161A>G	0.39±0.10	(-)	probably d.	tolerated	deleterious	deleterious	neutral	deleterious
rs45545238 p.Gln60His; c.180G>C	0.41±0.081	<0.001	probably d.	deleterious	deleterious	deleterious	effect	deleterious
rs772395988 p.Asp64Glu; c.192C>G	0.47±0.12	(-)	probably d.	tolerated	deleterious	deleterious	effect	deleterious
rs749132928 p.Asn78His; c.232A>C	0.23±0.075	(-)	probably d.	deleterious	deleterious	deleterious	neutral	deleterious
rs748381738 p.Tyr85Cys; c.254A>G	0.30±0.020	(-)	probably d.	deleterious	deleterious	deleterious	effect	deleterious
rs531591895 p.Arg95Trp; c.283C>T	0.64±0.070	<0.001	probably d.	deleterious	deleterious	deleterious	effect	deleterious
rs774890803 p.Arg101Ser; c.301C>A	0.44±0.060	(-)	probably d.	deleterious	deleterious	deleterious	effect	deleterious
rs776281553 p.Tyr102His; c.304T>C	0.45±0.097	(-)	probably d.	deleterious	deleterious	deleterious	effect	deleterious
rs143058517 p.Val111Met; c.331G>A	0.24±0.019	<0.001	probably d.	deleterious	deleterious	deleterious	effect	deleterious

rs776444658 p.Pro135Ser; c.403C>T	0.71±0.098	(-)	probably d.	deleterious	deleterious	deleterious	effect	deleterious
rs138676148 p.Arg139Gly; c.415A>G	0.31±0.040	(-)	benign	tolerated	deleterious	neutral	effect	neutral
rs761638904 p.Phe140Cys; c.419T>G	0.11±0.023	(-)	probably d.	deleterious	deleterious	deleterious	effect	deleterious
rs765352781 p.Phe140Leu; c.420C>G	0.31±0.010	(-)	probably d.	deleterious	deleterious	deleterious	effect	deleterious
rs143407371 p.Ala157Val; c.470C>T	0.65±0.038	(-)	benign	deleterious	neutral	neutral	effect	neutral
rs146238243 p.Pro159Leu; c.476C>T	0.27±0.10	(-)	possibly d.	deleterious	deleterious	deleterious	effect	deleterious
rs139424576 p.Asp167His; c.499G>C	0.52±0.073	(-)	probably d.	deleterious	deleterious	deleterious	effect	deleterious
rs777126816 p.Leu169Val; c.505C>G	0.72±0.090	(-)	probably d.	deleterious	deleterious	deleterious	effect	deleterious
rs143371936 p.Ala193Val; c.578C>T	0.63±0.078	<0.001	probably d.	deleterious	deleterious	deleterious	effect	deleterious
rs772557385 p.Cys195Tyr; c.584G>A	0.14±0.039	(-)	probably d.	deleterious	deleterious	deleterious	effect	deleterious

rs148373909 p.Arg207Cys; c.619C>T	0.12±0.022	0.004	probably d.	deleterious	deleterious	deleterious	effect	deleterious
rs755793031 p.Ile218Asn; c.653T>A	0.50±0.068	(-)	probably d.	deleterious	deleterious	deleterious	effect	deleterious
rs750842674 p.Ala226Gly; c.677C>G	0.72±0.043	(-)	benign	deleterious	deleterious	neutral	effect	neutral
rs200620452 p.Thr229Met; c.686C>T	0.21±0.037	(-)	probably d.	deleterious	deleterious	deleterious	effect	deleterious
rs767031004 p.Cys231Arg; c.691T>C	0.13±0.008	(-)	probably d.	deleterious	deleterious	deleterious	effect	deleterious
rs774941207 p.Val237Met; c.709G>A	0.51±0.041	(-)	probably d.	deleterious	deleterious	deleterious	neutral	deleterious
rs750084018 p.Gly240Arg; c.718G>C	0.23±0.041	(-)	probably d.	deleterious	deleterious	deleterious	effect	deleterious
rs1053874 p.Gln244Arg; c.731A>G	0.48±0.015	0.494	benign	tolerated	neutral	neutral	effect	neutral
rs8176939 p.Ala246Thr; c.736G>A	0.16±0.060	<0.001	possibly d.	deleterious	deleterious	deleterious	effect	deleterious
rs762211502 p.Asp265Tyr; c.793G>T	0.53±0.17	(-)	benign	tolerated	neutral	neutral	neutral	neutral
rs566266065	0.51±0.13	(-)	probably d.	deleterious	deleterious	deleterious	effect	deleterious

p.Glu278Ala; c.833A>C

SNPs elevating the activity

rs147546841<br>p.Tyr46His; c.136T>C	1.85±0.63	(-)	possibly d.	deleterious	deleterious	deleterious	neutral	neutral
rs140187838<br>p.Tyr46Ser; c.137A>C	2.65±0.43	(-)	possibly d.	deleterious	deleterious	deleterious	neutral	neutral
rs553922255<br>p.Gln79Arg; c.236A>G	1.40±0.11	<0.001	benign	tolerated	neutral	neutral	neutral	neutral
rs756148843<br>p.Val89Ile; c.265G>A	1.61±0.25	(-)	benign	tolerated	neutral	neutral	neutral	neutral
rs775559875<br>p.Asp115His; c343G>C	1.32±0.070	(-)	probably d.	deleterious	deleterious	deleterious	effect	deleterious
rs141801594<br>p.Asp120Asn; c.358G>A	1.87±0.40	<0.001	benign	tolerated	deleterious	deleterious	neutral	neutral
rs8176919<br>p.Gly127Arg; c.379G>A	2.11±0.52	0.022	probably d.	deleterious	deleterious	deleterious	effect	deleterious
rs755327078<br>p.Asn128Lys; c.384C>A	1.62±0.13	(-)	probably d.	tolerated	deleterious	neutral	neutral	neutral
rs139615062<br>p.Arg143Gln; c.428G>A	1.76±0.43	(-)	benign	deleterious	neutral	neutral	effect	deleterious
rs563863449	2.23±0.44	<0.001	benign	tolerated	neutral	neutral	effect	neutral

p.Arg148Ser; c.444G>T								
rs368307903<br>p.Glu149Gln; c.445G>C	2.53±0.22	(-)	benign	deleterious	neutral	neutral	effect	neutral
rs201942334<br>p.Ile166Met; c.498C>G	1.45±0.10	<0.001	benign	deleterious	deleterious	deleterious	neutral	neutral
rs150933932<br>p.Ala168Val; c.503C>T	1.64±0.31	<0.001	possibly d.	deleterious	deleterious	neutral	neutral	deleterious
rs199540176<br>p.Glu183Gln; c.547G>C	2.21±0.59	(-)	benign	tolerated	neutral	neutral	effect	neutral
rs751278286<br>p.Met186Ile; c.558G>A	2.22±0.15	(-)	benign	tolerated	neutral	neutral	neutral	neutral
rs760919591<br>p.Leu208Pro; c.623T>C	1.98±0.23	(-)	probably d.	deleterious	deleterious	deleterious	effect	deleterious
rs758781686<br>p.Thr213Ile; c.638C>T	1.61±0.13	(-)	benign	tolerated	neutral	neutral	neutral	neutral
rs774087461<br>p.Asp220Asn; c.658G>A	2.31±0.62	(-)	probably d.	tolerated	deleterious	deleterious	neutral	neutral
rs138354028<br>p.Ser221Asn; c.662G>A	1.86±0.23	(-)	benign	tolerated	neutral	deleterious	neutral	neutral
rs776953445<br>p.Leu263Val; c.787C>G	2.04±0.11	(-)	probably d.	deleterious	deleterious	neutral	effect	deleterious

a) The activity of each DNase I isoform was compared with that of the wild type using unpaired Student's *t* test; differences at $p < 0.05$ were considered to be statistically significant. Based upon the significant effect on the activity, SNPs could be classified into 4 categories.

b) The values are expressed as relative activity of each amino acid-substituted construct in the cell lyzates to that of the wild-type, representing the mean±SD ($n = 4$); n.d., the activity derived from the corresponding amino acid substituted construct could not be detected under our assay conditions.

c) Taken from the Ensembl database (https://asia.ensembl.org).

d) probably d., probably damaging; possibly d., possibly damaging
