# Supplementary material for: Evaluation of the functional effects of genetic variants‒missense and nonsense SNPs, indels and copy number variations‒in the gene encoding human deoxyribonuclease I potentially implicated in autoimmunity
Source: Sci Rep. 2019 Sep 20;9:13660. doi: 10.1038/s41598-019-49935-y (PMC6754452; doi:10.1038/s41598-019-49935-y)
Supplement: Supplementary file 3 — Supplementary table 4 [file 41598_2019_49935_MOESM3_ESM.pdf]

Evaluation of the functional effects of genetic variants–missense and nonsense SNPs, indels and copy number variations–in the gene encoding human deoxyribonuclease I potentially implicated in autoimmunity

Misuzu Ueki[1], Kaori Kimura-Kataoka[2], Junko Fujihara[2], Reiko Iida[3], Yasuyuki Kawai[4], Akari Kusaka[2], Takamitsu Sasaki[2], Haruo Takeshita[2*], Toshihiro Yasuda[1]

[1]Department of Medical Genetics and Biochemistry, Faculty of Medical Sciences, University of Fukui, Eiheiji, Fukui, Japan
[2]Department of Legal Medicine, Shimane University School of Medicine, Enya, Izumo, Japan
[3]Department of Life Sciences, Faculty of Medical Sciences, University of Fukui, Eiheiji, Fukui, Japan
[4] Department of Cardiology, Kanazawa Medical University, Uchinada, Ishikawa, Japan

*Corresponding author
E-mail: htakeshi@med.shimane-u.ac.jp (HT)

Supplementary table 4. **All the inframe insertion/deletion SNPs, together with nonsense mutations in *DNASE1* examined; effect of the corresponding amino acid substitution on the activity, prediction of the effect of the amino acid substitution on the activity using PROVEAN and global minor allele frequency (MAF)**

Indels/SNPs	Activity[a)]	Effect on the activity[b)]	Prediction by PROVEAN[c)]	Global MAF[d)]
rs121912990<br>p.Lys5*; c.13A>T	n.d.	abolishing	n.p.	(-)
rs768282770<br>p.delLeu10_Ala12; c.28_36delCTGCTGGCA	0.33±0.029	reducing	deleterious	(-)
rs565570420<br>p.Leu23_Lys24insLys; c.68_69insGAA	n.d.	abolising	deleterious	<0.001<br>mono-allelic[e)]
rs772295243<br>p.delGlu61; c.179_181delAGG	n.d	abolising	deleterious	(-)
rs775838249<br>p.delSer65_His66; c.191_196delACAGCC	n.d.	abolishing	deleterious	(-)
rs762443492<br>p. Asn76_Leu77insLysGluValArgAspSer; c.227_228insGGAGGTCAGAGACAG	0.21±0.18	reducing	deleterious	(-)
rs1436181412<br>p.Ser116_Tyr117insTyrTyrTyrAspAsp; c.347_348insCTACTACTACGATGA	1.20±0.51	not affecting	deleterious	(-)
rs1225420789<br>p.Tyr118*; c.354C>A	n.d.	abolishing	n.p.	(-)

rs749069636	0.97±0.12	not affecting	deleterious	(-)
p.delAsp120; c.358_360delGAT				
rs775542282	0.91±0.093	not affecting	deleterious	(-)
p.delAsp129; c.383_385delACG				
rs745357597	n.d.	abolishing	n.p.	(-)
p.Arg133*; c.397C>T				
rs1428191765	0.55±0.11	reducing	deleterious	(-)
p.delPhe140; c.418_420delTTC				
rs766818831	n.d.	abolishing	deleterious	(-)
p.Ile166_Asp167insAspAlaLeuTyrVal; c.493_494insTCGACGCTCTCTATG				
rs13463995128	n.d.	abolishing	deleterious	(-)
p.Asp184_Val185insVal; c.550_551insCGT				
rs751121067	n.d.	abolishing	deleterious	(-)
p. delSer196; c.584_586delGCA				
rs753840602	n.d.	abolishing	n.p.	(-)
p.Trp209*; c.626G>A				
rs776775847	n.d.	abolishing	deleterious	(-)
p.Arg235_Ile236insIleVal; c.705_706insGATCGT				
rs773837277	n.d.	abolishing	deleterious	(-)
p.delAsn256_Ala260; c.765_779delTAACTTCCAGGCTGC				
rs201571412	n.d.	abolishing	n.p.	(-)

p.Gln244*; c.730C>T				
rs749480593<br>p.Gln258*; c.772C>T	n.d.	abolishing	n.p.	(-)
rs764966194<br>p.Gln269*; c.805C>T	n.d.	abolishing	n.p.	(-)
rs753724955<br>p. Ile271_Ser272insSerAspHisTyrPro; c.813_814insAGTGACCACTATCCAGT	n.d.	abolishing	deleterious	(-)

a) The values are expressed as relative activity of each construct in the cell lyzates to that of the wild-type, representing the mean±SD (n =4); n.d., the activity derived from the corresponding construct could not be detected under our assay conditions.

b) Based upon the effect on the activity, SNPs could be classified into 4 categories.

c) Effect of the inframe insertion/deletion corresponding to each variant on the activity was predicted using PROVEAN; n.p., not predicted.

d) Taken from the Ensembl database (http://asia.ensembl.org/Homo_sapiens/).

e) Genetic heterogeneity in our study populations is shown.
